# Use of Cognitive Task Analysis to Understand Decision-making for Management of Blunt Abdominal Trauma in Children

**DOI:** 10.7759/cureus.4095

**Published:** 2019-02-19

**Authors:** Tania Ahluwalia, Serkan Toy, Chris Kennedy

**Affiliations:** 1 Emergency Medicine, Children's Mercy Hospital, Kansas City, USA; 2 Anesthesiology, Johns Hopkins University School of Medicine, Baltimore, USA

**Keywords:** abdominal trauma, cognitive task analysis, decision-making

## Abstract

Study objective

To use cognitive task analysis (CTA) to elicit experts’ knowledge and outline their clinical decision-making process for the management of pediatric blunt abdominal injury.

Methods

This was a mixed-methods study involving in-depth interviews and a focus group with local experts followed by a review survey with a panel of experts from numerous pediatric trauma centers across the United States. All experts specialized in pediatric emergency medicine and pediatric trauma surgery.

Results

Common themes that emerged during seven in-depth interviews with local experts included: clinical management, clinical reasoning, situational awareness, potential errors/novice traps, knowledge and skills, communication, and quality indicators. A total of 17 external experts responded to the survey for a response rate of 77%. External experts indicated that the information outlined in the CTA was complete and accurate, and provided valuable insights into the discrepancies that were unresolved during the focus group with local experts. They indicated agreement with potential errors/novice traps reported by local experts. Specifically, they indicated that the failure in coordinating team activities, maintaining the big picture, and performing a thorough physical examination posed serious threats to novices when managing a child with blunt abdominal trauma.

Conclusion

CTA may be applied to pediatric management of blunt abdominal injury to identify critical steps and potential errors to serve as a framework for education in pediatric emergency medicine and pediatric trauma surgery. Future studies may apply CTA to other types of traumatic injuries and/or involve an interdisciplinary approach.

## Introduction

Background

Injury is the leading cause of death in children from ages 1 to 18 years [1]. The severely injured child demands prompt medical intervention for an optimal outcome. Since 80% of children are first cared for by emergency medical services (EMS) and in community hospitals, these healthcare systems must be prepared [2]. Caring for the injured child presents unique challenges for healthcare providers. For example, to detect derangements providers must be familiar with normal values for vital sign ranges, which change with age and are significantly different from adults. Unlike adults, the early recognition of shock in children can be very challenging. An increased heart rate may be the only sign of shock. However, there are numerous other causes of increased heart rate in children, such as pain or anxiety, which confound decision-making. Additionally, treatments and interventions require child-specific decisions be made, in terms of equipment, medication dosage, and definitive care. To remedy this situation, practitioners need an avenue to learn about the management of pediatric trauma patients, specifically to improve their decision-making skills and to reduce the risk of potential errors. Training focused on modeling expert performance can help enhance a practitioner’s ability to manage emergent trauma cases. For this complex task, simply observing expert behaviors is not enough. It is crucial to understand the cognitive decisions that underlie expert actions [3]. Decision-making for management of traumatic patients is second-nature for experts, and it may be challenging for them to provide a detailed account of their thought process.

Importance

Cognitive task analysis (CTA) is a method to elicit expert knowledge by dissecting a task or skill into critical steps and by identifying the associated thought processes. This method helps uncover the cognitive processes underlying decision-making that are not accessible to direct observation [4-7]. In other words, CTA is a way to understand how an expert thinks by identifying cognitive activities needed to complete a task. The insights into experts’ decision-making processes may provide a useful framework for teaching and learning. CTA has been used to gain a deeper understanding of the thought processes applied by surgical subspecialists while performing specific procedures such as endoscopic retrograde cholangiopancreatography, vaginal hysterectomy, central venous placement, and laparoscopic procedures [5,6,8-10]. Data from these studies led to the development of procedural checklists for use in training and assessment.

Goals of this investigation

Acute trauma management presents as an ideal clinical scenario to apply CTA to assist practitioners in making life-altering decisions regarding management of the traumatically injured child. We sought to use the CTA methodology to gain a more thorough and detailed understanding of the decision-making process for managing acute pediatric blunt abdominal trauma, the most common unrecognized fatal injury and the third most common cause of pediatric trauma mortality [11].

## Materials and methods

Study design and setting

This study obtained exempt status from the local institutional review board. The design and reporting of this study were based on the consolidated criteria for reporting qualitative research (COREQ) guidelines, a 32-item checklist to report key characteristics of qualitative research studies [12]. This was a mixed-methods study conducted in two phases. The first phase was a qualitative study involving in-depth interviews and a focus group with local experts, which aimed to develop a deeper understanding of the expert clinical decision-making process for managing pediatric abdominal trauma patients. In the second phase, we employed a review survey with a panel of experts from numerous pediatric trauma centers across the United States to triangulate the results that emerged in the qualitative study.

Phase I – Qualitative study/selection of participants

Semi-structured interviews were conducted with local experts at a single site, a pediatric regional trauma center in the Midwest with an annual census of 75,000. A total of seven experts (four pediatric emergency medicine (PEM) attending physicians, two pediatric trauma surgeons and one pediatric trauma surgery fellow with prior experience as a surgeon) from our institution were recruited to participate in this study. Experts were defined as board-certified PEM attendings or board-certified surgeons with >5 years of experience in management of pediatric blunt abdominal trauma, experience as trauma team leaders, involvement with graduate medical education as fellowship directors or as preceptors for trainees, and/or involvement as active members of trauma committees at local or national levels.

Data were collected during a private audio-recorded interview with each expert. Interview length was a maximum of 1.5 hours. Experts were interviewed separately to avoid premature consensus building. Standardized interviews were developed based on evidence-based literature including Advanced Trauma Life Support (ATLS) course [13] and a published clinical pathway for managing pediatric patients with blunt abdominal trauma [11]. Interviews were semi-structured to provide organization for the interviewer and allow the experts flexibility to explore their clinical decision-making processes beyond the standard interview protocol when necessary. Interview protocol included two clinical vignettes to gain insight into experts’ internal thought processes when managing a child with blunt abdominal trauma (refer to Table 1). Medical information related to each case was revealed gradually to simulate the dynamic nature of real-life information gathering and to expose experts’ evolving decision-making processes. The interviewers asked the experts probing questions regarding the thought processes behind their clinical decisions (including the timing, and order or sequence of management steps) as they explained how they would manage each of the pediatric blunt abdominal trauma cases. Each expert was prompted with open-ended interview questions to explore critical decision points, potential errors/novice traps, helpful sensory cues, necessary knowledge and skills, and quality indicators that informed their management of pediatric blunt abdominal trauma. Key open-ended questions used during the interviews are listed in Table 2.

**Table 1:** Clinical Vignettes. ED: Emergency department; EMS: Emergency medical services

Case	Clinical Vignette
Case 1	In the ED, you receive a phone call “We have an 11-year-old female who was in a motor vehicle accident, she was restrained and is complaining of abdominal pain. Vital signs: heart rate 130 beats/min, blood pressure 105/70 mmHg, respiratory rate 20 breaths/min, and oxygen saturation 98% on room air. Estimated time of arrival: 5 min”. EMS arrives and report that she was restrained in the rear driver’s-side seat of the vehicle with a lap and shoulder belt when the vehicle was struck at high speed on the driver’s side. On arrival to the ED, she is awake and alert, immobilized with a cervical collar. Her vital signs are: temperature 37.5, vital signs as reported previously. She is able to maintain her airway and has clear and equal breath sounds without increased work of breathing. She has strong distal pulses. She complains of abdominal pain. Her abdomen is soft and non-distended but she has localized tenderness in the left upper quadrant. There are no bruises or abrasions noted on the abdomen.
Case 2	As you are managing your case, the charge nurse notifies you that “the colliding car has a 12-year-old patient who will arrive in 5 minutes. He was restrained in the rear passenger’s side seat of the vehicle with a lap and shoulder belt when the vehicle was struck at high speed on the driver’s side. He has a cervical collar in place” [Note: you are the only attending in the ED]. His vital signs are: temperature 37.4, heart rate 135 beats/min, blood pressure 80/50 mmHg, respiratory rate 20 breaths per minute and oxygen saturation 99% on room air. On arrival to the ED, vitals as mentioned previously, he is awake and alert, immobilized with a cervical collar. His airway is patent and he has clear and equal breath sounds bilaterally without increased work of breathing. He has weak distal pulses and his capillary refill is 4 seconds. He complains of abdominal pain and left shoulder pain. On examination, his abdomen is soft and non-distended but he has localized tenderness in the left upper quadrant. He has no bruises or abrasions noted on the abdomen. His genitourinary examination is unremarkable.

**Table 2:** Key Interview Questions.

Case	Questions
Cases 1 & 2	How would you prepare for this patient? What are your specific clinical goals and objectives when managing this patient? What would you do first? How did any information from the initial assessment change your clinical management? What concerns or questions are running through your mind about this patient? What parts of your clinical assessment led you to determine which labs you would order? How do you determine what imaging this patient needs? What other courses of action were considered or available? What mistakes are likely at this point? How might a novice behave differently? If you were asked to describe the situation to a colleague at this point, how would you summarize the situation? What is the disposition for this patient? What were key decisions you had to make while managing this case? If you were notified [about case 2] at the same time of arrival of your first patient, how would you prepare for this patient? How would you triage this patient? How would you co-manage this case? At what point would you leave the first patient to attend to this patient? When and how did you know this was blunt abdominal trauma? Do you think a novice could have missed this?
Previous Case Questions	Could you tell me about a typical case of blunt abdominal trauma with hemorrhage? What were your assessment and treatment priorities for your patient? How much time pressure was involved in making decisions? What quality indicators were used to successfully manage this patient? What kind of sensory cues were helpful in your decision-making process? Does this case fit a case you were trained to deal with? What specific training or experience was necessary or helpful in this decision? Can you recall one of the first patients you took care of with pediatric blunt abdominal trauma? How has your experience from a medical student/intern to an experienced attending changed your management of this type of patient? What other training, experience, knowledge or information would have been useful? Can you tell me about the last case you encountered with a patient presenting with blunt abdominal trauma with hemorrhage? What pitfalls did you note when observing a fellow or resident lead a case of blunt abdominal trauma? Have you had a referral from an outside hospital with blunt abdominal trauma? What are the challenges when accepting these cases/with these transfers? Do you know while you are taking the call that the case is blunt abdominal trauma? What are the clues? If you have to teach novice in 10 minutes how to manage a case of pediatric blunt abdominal trauma what key decision-making points would you teach them?

The interviews took place in the hospital setting in a private conference room, during the workday, and without interruptions. Each participant provided written consent for this voluntary activity. The principal investigator (T.A.), a second-year fellow in PEM, conducted the interviews after receiving training in cognitive task analysis by a professionally trained instructional designer/educational researcher (S.T.) and co-leading a pilot interview with S.T. T.A. was present at all interviews, S.T. co-led six interviews and C.K. co-led one interview. Audio recordings of the interviews were transcribed by a medical transcriber and reviewed by the investigators for accuracy. The audio recordings contained no participant personal identifiers, such as the name or title of the participant. Audio recordings were deleted upon completion of the study. No repeat interviews were required.

Analysis

The written transcriptions were analyzed using a hybrid thematic analysis approach [14]. This hybrid method, combining deductive and inductive thematic analysis, allowed the researchers to use the existing evidence base on medical decision-making/clinical-problem solving and the published clinical pathway for managing pediatric patients with blunt abdominal trauma as a framework to create a coding scheme [5,15-17]. This hybrid methodology was flexible to extend the coding scheme beyond the initial framework based on the data to account for more task-specific and covert expert cognitive reasoning processes underlying the observable skills that have not been addressed in the literature previously [16]. See Table 3 for the coding scheme.

**Table 3:** Coding Scheme. * Refer to “Clinical Pathway for Management of the Pediatric Patients with Blunt Abdominal Trauma” [11] FAST: Focused assessment with sonography for trauma

Theme	Sub-Themes
Situation Awareness	Gather initial information, use sensory cues, take anticipatory steps (prepare team/equipment), assess situation, maintain big picture, activate trauma/trauma consult, triage patients, prioritize tasks, remain vigilant
Clinical Reasoning	Review data, interpret data, order relevant tests, order imaging, consider hypothesis
Clinical Management*	Perform primary survey, obtain intravenous access, give fluids as needed (crystalloid), blood products/massive transfusion protocol, perform FAST, perform secondary survey, form goals, revise management based on change/new information, pain management, co-manage multiple patients, re-evaluate patient, disposition (discharge/operation room/admit), critical steps
Knowledge, Skills & Attitudes	Protocols, algorithms, procedural skills, physical exam, experience, concepts, training, desire to improve
Communication & Teamwork	Exchange information, establish shared understanding, communicate goals, delineate tasks, coordinate team activities, referral, trust
Potential Errors/Novice Traps	Tunnel vision/fixation/anchoring, communication, coordinating team activities, maintaining big picture, interpreting vital signs and recognizing shock, omission bias, thorough physical examination, missing sensory cues, re-evaluate, prioritizing tasks
Quality Indicators & Safety Measures	Speed, system-based care, standardized care, optimizing number of team members

The transcribed interviews were analyzed using the qualitative data analysis software, NVivo for Mac (QSR International Pty Ltd., Version 11. 3.2, 2016, Melbourne, Australia). Two researchers (T.A. and S.T.) independently coded the transcribed interviews using the above-mentioned coding scheme. The data led to the development of sub-themes. Identified themes were compared and coding disagreements were resolved by an iterative process with consensus. A third researcher (C.K.) resolved any remaining minor disagreements. Data obtained from the coded transcriptions were combined into the final set of themes and discrepancies (observed among experts) representing the experts’ cognitive reasoning processes for managing pediatric abdominal trauma cases.

The data from the interviews were reviewed by the participating internal experts during a 90-minute focus group session with the aim of reviewing the collected data and discussing discrepancies with the experts to understand their rationale for decision-making. The focus group included five of the seven internal experts. The session was facilitated by two researchers (T.A. and C.K.). It was audio-recorded in the hospital setting. This served as a member check to ensure the accuracy of qualitative data.

Phase II – External expert survey

Researchers suggest that triangulation helps strengthen the reliability and validity of a qualitative study [18-20]. Certain policies, regulations, and practices specific to the local study site could have influenced the clinical decision-making processes that emerged in the qualitative phase. Therefore, as the last iteration of the CTA, external experts in pediatric trauma across the United States were recruited to review the cognitive task analysis report in the form of a survey.

Selection of participants

A total of 11 PEM attendings and 11 pediatric trauma surgeons from other facilities were identified through peer nomination. They all met the above inclusion criteria for experts and were invited to participate in the external expert review. The participating experts represented diverse geographical locations across the United States. Experts were contacted via email and each expert received one reminder to complete the survey. An online expert review survey was created using Qualtrics software (http://www.qualtrics.com, Qualtrics, Provo, UT). The survey items were based on the key findings and discrepancies related to management of pediatric blunt abdominal trauma highlighted in the first phase of the study (see Table 4 for key questions in the survey for external experts). External experts did not have access to identifiable information. Their role was to review the key findings from the interviews and focus group and to help resolve any discrepancies in the form of a survey. The anonymity of external experts was maintained.

**Table 4:** Key Question Topics in the Survey for External Experts.

Key Questions
Timing for completion of primary survey
Timing for completion of secondary survey
Pre-arrival steps in management of a child with suspected blunt abdominal trauma
Steps for management of a child with suspected blunt abdominal trauma
Laboratory tests to order
Factors to consider before pain management
Timing of chest X-ray
Use of focused assessment with sonography in trauma (FAST) examinations
Use of pelvic X-rays
Need for volume resuscitation
Type of volume resuscitation
Occurrence of potential errors in trauma care

Analysis

The external expert survey data were exported to Microsoft Excel (Microsoft, Redmond, WA) for analyses. Categorical data were presented in frequencies (and percentages), and numerical data were provided as means (standard deviations and 95% confidence intervals). For the ordinal data related to time intervals of management steps, we converted each time interval to a numerical value (i.e., > 0 to < 1 minute = 1; > 1 to < 5 minutes = 2, etc.) and calculated the mean, standard deviation, median, and mode based on this conversion. Examination of all three central tendency measures helped determine the corresponding time interval by which each of the management steps should be completed.

## Results

Phase I – Qualitative data analysis results

Interviews were completed with seven experts. The median interview time was 56 minutes 48 seconds (range 32:57-83:10) with a median word count of 7,917 (range 5,559-14,665) and a total sum of 31,686 words. Common themes were described in the coding scheme and the frequencies and relative frequencies from each sub-theme are listed in Tables 5-11. Table 12 shows representative quotations pertinent to each theme.

**Table 5:** Sub-themes for Clinical Management. FAST: Focused assessment with sonography for trauma

Clinical Management	Frequency	Relative Frequencies
Give crystalloid fluids as needed	35	15.49
Perform primary survey	34	15.04
Perform secondary survey	28	12.39
Re-evaluate patient	22	9.73
Pain management	18	7.96
Perform FAST examination	17	7.52
Disposition	15	6.64
Intravenous access	13	5.75
Blood products, massive transfusion protocol	12	5.31
Revise management based on change or new data	11	4.87
Form goals	10	4.42
Co-manage multiple patients	8	3.54
Remain vigilant	3	1.33

**Table 6:** Sub-themes for Clinical Reasoning.

Clinical Reasoning	Frequency	Relative Frequencies
Order imaging	50	29.76
Consider hypothesis	44	26.19
Review and interpret data	38	22.62
Order relevant tests	36	21.43

**Table 7:** Sub-themes for Situational Awareness.

Situational Awareness	Frequency		Relative Frequencies
Trauma activation or consult		38	25.17
Gather initial information		35	23.18
Anticipatory step		22	14.57
Triage patients		14	9.27
Assess situation		13	8.61
Prioritize tasks		11	7.28
Use sensory cues		10	6.62
Maintain big picture		5	3.31
Remain vigilant		3	1.99

**Table 8:** Sub-themes for Potential Errors & Novice Traps.

Potential Errors & Novice Traps	Frequency	Relative Frequencies
Tunnel vision	18	25.71
Incomplete physical examination	15	21.43
Maintaining big picture	11	15.71
Recognizing shock	10	14.29
Omission bias	5	7.14
Coordinating team activities	4	5.71
Missing sensory cues	3	4.29
Communication	2	2.86
Prioritizing tasks	2	2.86

**Table 9:** Sub-themes for Knowledge & Skills.

Knowledge & Skills	Frequency	Relative Frequencies
Experience	21	31.34
Training	15	22.39
Protocols	14	20.90
Physical exam	13	19.40
Algorithms	2	2.99
Desire to improve	1	1.49
Procedural skills	1	1.49

**Table 10:** Sub-themes for Communication & Teamwork.

Communication & Teamwork	Frequency	Relative Frequencies
Establish shared understanding	17	29.82
Coordinate team activities	16	28.07
Exchange information	10	17.54
Communicate goals	6	10.53
Delineate tasks	5	8.77
Trust	3	5.26

**Table 11:** Sub-themes for Quality Indicators.

Quality Indicators	Frequency	Relative Frequencies
Speed	30	68.18
System-based care	11	25.00
Optimizing number of team members	1	2.27
Standardized care	1	2.27
Written orders	1	2.27

**Table 12:** Additional Representative Quotations from Cognitive Task Analysis. ATLS: Advanced Trauma Life Support

Theme	Quotations
Clinical Management	“Everybody is coming from the same knowledge base [ATLS]…if we’re all talking about it in the same way – same vocabulary, same strategy of evaluating the patient…then you have a common way of discussing what’s next… that’s a key way of…educating those that are going to be… front line or a quaternary referral center...then we have a much better chance of delivering quality care from the beginning to the end.”
Clinical Reasoning	“The [transferring physician may] say, ‘this kid’s got some sort of abdominal injury,” but they might not describe the child as in shock…you kind of have to infer from the information that they’re giving you that this kid’s sick or not sick.” Regarding Case 2 “he has pain in the shoulder and in the left upper quadrant, which…means everything in between potentially…he could have chest injury…a clavicle fracture and a spleen injury or a bruised belly. But given his vital signs, it makes me even more worried”.
Situational Awareness	“I try to think about what do I know about the patient. And based on what we know, what is most likely, and then what else might be there that we’re not expecting”.
Potential Errors/Novice Traps	Refer to results phase I: qualitative data analysis results section of the manuscript
Knowledge, Skills and Attitudes	“ATLS or any type of trauma algorithm helps remove a lot of uncertainties so everybody knows what the next step is” [subtheme: algorithm].
Communication and Teamwork	“… in educating our trainees - we have to teach them to speak up and include everybody else”
Quality Indicators and Safety Measures	Refer to results phase I: qualitative data analysis results section of the manuscript

Clinical Management

Experts shared their decision-making steps to manage a child with blunt abdominal injury. They stated the importance of triaging patients, even before the patient arrives via EMS, by listening for sensory cues, such as, listening to the tone of the EMS provider and the sound of sirens, and interpreting vital signs based on age when deciding to activate a trauma. Experts emphasized the importance of forming goals, such as performing a quick yet thorough primary examination with emphasis on managing the patient’s airway and hemodynamic stability followed by conducting a systematic secondary survey. An expert emphasized the importance of teaching critical steps to manage traumatically injured children, “I teach every resident the same thing – a head to toe evaluation, whether they come in with abdominal pain, leg pain etc…the key in all traumas in the ER is your initial overall survey” [sub-theme: perform primary survey; perform secondary survey]. Experts also highlighted the importance of giving crystalloid fluids as needed and re-evaluating the patient after each intervention. Moreover, experts discussed the challenges associated with co-managing trauma patients, such as mixing up orders, labs, or medications. Experts noted the importance of re-evaluating multiple patients without minimizing the patient that initially did not present as sick as the other patient. Experts discussed the benefits of training and the importance of establishing a shared standard vocabulary when evaluating the patient. There were discrepancies among experts about the use of focused assessment with sonography for trauma (FAST) examinations and use of pelvic X-rays.

Clinical Reasoning

Experts offered a detailed account of their thought processes for ordering tests and imaging studies, hypotheses, as well as, reviewing and interpreting data. An expert revealed that “my decision point in terms of choosing modality of imaging is based on the clinical exam and lab values” [sub-theme: order imaging]. Another expert acknowledged the importance of reviewing and interpreting data when a patient’s care is transferred between providers or between facilities. He stated that a key decision-making point is deciding if the child is sick or not sick. Also regarding patient transfers an expert specified the importance of being “mindful of what labs you need to repeat, what has been done, what’s available to you, is the imaging of any good quality” [sub-theme: review and interpret data].

Situational Awareness

This refers to identifying, processing and comprehending information to anticipate subsequent steps. It is essential for a trauma team leader to have situational awareness in order to prepare the critically injured child and the team for the next steps in management. The most common sub-themes that emerged for situational awareness included trauma activation/consult, gathering initial information and anticipatory steps. Additional sub-themes included: triaging patients, assessing the situation, prioritizing tasks, using sensory cues, maintaining the big picture, and remaining vigilant. An expert stated that “it’s best if you can teach trainees to be hands off at a 30,000 foot view – looking at everything that’s happening” to consolidate information and maintain the big picture in order to make key decisions in management. Additionally, an expert emphasized the importance of prioritizing tasks while managing a critically injured child. The expert questioned “what’s going to kill the patient and what’s going to save the patient?…A shoulder injury is not going to kill the patient”.

Potential Errors/Novice Traps

Experts thought that tunnel vision, incomplete physical examination, and failure to maintain big picture could pose as some of the most common potential errors in the management of a pediatric trauma patient. An expert stated that “with novices it’s hard to ignore the obvious - if blood and guts are hanging out, they focus on that” [sub-theme: tunnel vision, fixation and anchoring]. Another expert described a novice trap as “trying to give multiple diagnoses…it’s easy to say, “Okay, it’s probably a clavicle injury and a spleen” and not thinking about, how can abdominal pain make your shoulder hurt?” [sub-theme: tunnel vision, fixation and anchoring]. Another expert stated that “90% of where people will mess up, is…going out of order, not being systematic…head to toe... when the person doing the evaluation goes off of that standard, things get lost, they don’t know where they left off, they don’t know what they’ve examined, they don’t know if they have looked at the chest or the back or they get confused. And once the leader gets confused, then everyone’s confused” [sub-theme: incomplete or non-systematic physical examination]. Another potential error is delayed recognition of abnormal vital signs and shock. An expert specified, “one of the biggest things we notice in trauma reviews is a delayed recognition of shock” and “tachycardia is one of the biggest things missed by referrals.” Additional potential errors include omission bias, failure to coordinate team activities, missing sensory cues, difficulty with communication and failure to prioritize tasks.

Knowledge, Skills, and Attitudes

Experts commented that experience, training, as well as, knowledge and adherence to protocols are crucial for the effective management of pediatric trauma cases. They also emphasized the importance of proficiency in physical examination, algorithms, continual practice improvement, and procedural skills. Regarding the sub-theme of experience, an expert stated, “we see so many injured children that…we’re just used to what their physiology is, what kind of work up needs to be done, as opposed to people that maybe only see one injured child in a whole year or career”.

Communication and Teamwork

Key components for communication and teamwork include establishing shared understanding, coordinating team activities and exchanging information. Additional components include communicating goals, delineating tasks, and creating an atmosphere of trust. For communication and teamwork, experts expressed that “the biggest area of danger is communication issues or misunderstandings” [sub-theme: establish shared understanding]. Another expert emphasized the importance of coordinating team activities [sub-theme: direct the rest of the people in the room, “okay, I need X, Y, Z done”].

Quality Indicators and Safety Measures

Experts agreed that speed is crucial in the management of a traumatically injured child. Time to complete the primary and secondary survey, time to get the patient to the computed tomography (CT) scanner and time to disposition are priorities. Other factors identified included systems-based care, optimizing the number of team members, performing standardized care and using written orders instead of verbal commands.

Focus Group with Local Experts

Experts reviewed the information collected during the interviews and confirmed their perspectives were captured accurately. Discrepancies among experts were discussed and mostly resolved during the focus group. Unsettled discrepancies included the timing of primary and secondary survey, the timing of chest X-ray (CXR), the utility of pelvis X-ray and use of FAST examinations. Internal experts at the focus group stated the primary survey should take between 30 seconds to 5 minutes and the secondary survey should be completed in 10 minutes. Some experts felt that the CXR should occur with the primary survey, whereas others thought it should take place during the secondary survey. Some experts stated that pelvic X-rays may not be useful, particularly if the patient gets a CT scan of the abdomen and pelvis. Regarding FAST exams, some experts questioned its utility if the patient will ultimately get a CT abdomen/pelvis or require transfer to the operating room. Some experts stated that FAST exams are helpful to evaluate for cardiac tamponade; however, they may not help with patient disposition such as surgical intervention.

Phase II – External expert review survey results

A total of 17 experts (out of 22) across the United States responded to the survey for a response rate of 77%. The external experts indicated that the information outlined in the first phase of the study was representative of the expert clinical decision-making process for managing pediatric blunt abdominal trauma cases. They also provided valuable insights into the discrepancies that were not resolved during the focus group with local experts.

The external experts recommended that the pre-arrival events should occur in the sequence listed in Table 13. Some of these steps could occur concurrently and the order could be adjusted for other cases. All experts agreed that these events should take place before the child arrives at the emergency department.

**Table 13:** Sequence of Pre-arrival Steps Ranked by External Experts (N = 17). *Lower score indicates priority. Weighted score is the sum of all the rankings provided by experts for each pre-arrival step. EMS: Emergency medical services

Pre-arrival Steps	Overall Rank	Rank Distribution – Frequency (%)						Weighted Score*
		1st	2nd	3rd	4th	5th	6th	
Upon EMS call, determine if the child is sick based on the vital signs, the mechanism and obvious injuries	1	14 (88)	1 (6)	0 (0)	1 (6)	0 (0)	0 (0)	20
Activate trauma team/process if appropriate	2	0 (0)	10 (63)	3 (19)	2 (12)	1 (6)	0 (0)	42
Establish a team leader and assign roles	3	1 (6)	0 (0)	5 (31)	7 (44)	2 (13)	1 (6)	60
Communicate requirements for staff, equipment & resuscitation	4	0 (0)	1 (6)	5 (31)	4 (25)	4 (25)	2 (13)	65
Obtain information from transport about mechanism	5	1 (6)	4 (25)	2 (12)	0 (0)	2 (12)	7 (44)	67
Estimate weight of patient	6	0 (0)	0 (0)	1 (6)	2 (12)	7 (44)	6 (38)	82

During the internal focus group, there were discrepancies regarding the duration of the primary and secondary survey. On average, external experts indicated that the primary survey should be completed within 2 minutes (SD = 1.36) with 95% CI [1.31, 2.61] and secondary survey within 5 minutes (SD = 2.99) with 95% CI [3.51, 6.25]. The experts also responded to a question about the time intervals within which critical management steps should be completed. All three central tendency measures (mean, median, and mode) were reasonably similar for each management step, which helped determine the corresponding time interval (see Table 14 for a complete list of management steps, and corresponding time intervals).

**Table 14:** External Expert Survey Summary Table for Timing of Management Steps (N = 17). *Following codes for time intervals were used for the statistical analysis: >0 to <1 = 1; >1 to <5 = 2; >5 to <15 = 3; >15 to <30 = 4; >30 to <60 = 5; >60 =6 CT: Computed tomography; ED: Emergency department; EMS: Emergency medical services; FAST: Focused assessment with sonography for trauma

Management Steps	Mean (SD)	Median	Mode	Corresponding Time interval*
Assess airway	1.24 (0.44)	1	1	>0 to <1 minute
Transfer patient from EMS gurney to ED bed	1.29 (0.47)	1	1	>0 to <1 minute
Apply monitors for heart rate, blood pressure, oxygen saturation	1.47 (0.62)	1	1	>0 to <1 minute
Apply high flow oxygen face mask, if needed	1.47 (0.51)	1	1	>0 to <1 minute
Assess breathing	1.53 (0.51)	2	2	>1 to <5 minutes
Assess circulation	1.53 (0.51)	2	2	>1 to <5 minutes
Assess disability	1.71 (0.59)	2	2	>1 to <5 minutes
Apply cervical collar, if needed	1.71 (0.69)	2	2	>1 to <5 minutes
Obtain intravenous access	2.00 (0.50)	2	2	>1 to <5 minutes
Identify the need for fluid resuscitation and consider need for blood products or massive transfusion protocol	2.06 (0.57)	2	2	>1 to <5 minutes
Expose the patient	2.12 (0.70)	2	2	>1 to <5 minutes
Team leader summarizes primary assessment	2.12 (0.70)	2	2	>1 to <5 minutes
Provide warming measures	2.47 (0.51)	2	2	>1 to <5 minutes
Obtain laboratory testing	2.53 (0.51)	3	3	>5 to <15 minutes
Roll patient and assess	2.53 (0.51)	3	3	>5 to <15 minutes
Assign a team member to update family and obtain further history	2.53 (0.87)	3	3	>5 to <15 minutes
Team leader provides repeated assessment stated out loud	2.59 (0.62)	3	3	>5 to <15 minutes
Consider pain management	2.71 (0.69)	3	3	>5 to <15 minutes
Perform a thorough head-to-toe assessment	2.82 (0.60)	3	3	>5 to <15 minutes
Consider FAST examination	3.00 (1.17)	3	3	>5 to <15 minutes
Consider CT abdomen/pelvis	3.06 (0.57)	3	3	>5 to <15 minutes
Obtain pelvic X-ray	3.41 (1.06)	3	3	>5 to <15 minutes
Determine disposition	3.94 (0.83)	4	4	>30 to <60 minutes

Based on the external experts’ responses, laboratory tests for a hemodynamically unstable patient (refer to Table 1, Case 2) should include: complete blood count (n = 17, 100%); liver function tests (n = 13, 76%); lipase (n = 13, 76%); type and screen (n = 10, 59%); basic metabolic profile (n = 10, 59%); urine dip (n = 9, 53%); urinalysis (n = 9, 53%); and type and cross (n = 9, 53%). Additional labs suggested by external experts included a coagulation panel, blood gas, and lactic acid.

Before deciding on pain management, experts considered vital signs (n = 17, 100%); weight (n = 15, 88%); past medical history (n = 10, 59%). Additional considerations before ordering pain medications included the patient’s mental status, signs of traumatic brain injury, allergy status, and hemodynamic status.

There were discrepancies among our internal experts regarding imaging studies, specifically, the timing of CXR, use of FAST exams and use of pelvic X-rays. Thirteen (76%) of the experts agreed that the CXR should be obtained after the primary survey, mostly stating that it should be obtained during the secondary survey after rolling the patient. Experts emphasized that the CXR should not delay resuscitation. Regarding FAST examinations, most external experts, 12 (71%), stated that ultrasound is useful to diagnose cardiac tamponade. Over half of the experts, nine (53%), stated that FAST examinations are helpful in detecting free fluid; however, CT abdomen/pelvis reveals more information. Eight experts stated that FAST exams are helpful for evaluating whether hemodynamically unstable patients should go to the operating room. Nearly one-third of the external experts, five (31.25%), stated that there are no contraindications to performing a FAST examination. A few experts, three (29%), thought that FAST examinations are not helpful in the disposition of a patient. Experts commented that FAST examinations contribute a data point to be combined with other factors. An expert mentioned that in hemodynamically unstable patients, FAST exams are helpful in determining where to start the operation: abdomen, heart, head or chest. Another expert commented that FAST exams are not useful for stable patients and another expert stated that there is no definite information on pediatric FAST examinations yet. Our data suggest that further studies on the utility of pediatric FAST examinations are needed.

Regarding the pelvic X-ray for the second patient, the majority of experts, 14 (82%), said they would consider obtaining a pelvis X-ray if there was a high-speed mechanism of injury in a patient who is hypotensive and has abdominal pain, to rule out a major source of bleeding, if the patient is unstable for CT due to an unstable pelvic fracture. Other experts commented that they would not obtain a pelvic X-ray unless the pelvis was unstable on exam or if they were obtaining a CT abdomen/pelvis.

As to volume resuscitation, 15 (88%) experts agreed that the patient in case 2 needed volume resuscitation. They rationalized that the patient was in hypovolemic shock (tachycardic with delayed capillary refill and weak pulses). The majority of experts, 11 (65%), agreed that the fluid volume resuscitation should be normal saline or lactated ringer’s 20 cc/kg. Experts commented that they would follow this with blood. One expert stated that it would be ideal to start with blood, but the delay in obtaining blood from the blood bank obliges the physician to start with normal saline or lactated ringer’s solution. Another expert suggested that crystalloid may contribute to coagulopathy. Experts commented that they would activate the massive transfusion protocol.

The potential errors/novice traps reported by local experts were validated by the external experts. Overall, external experts indicated that the following errors could occur commonly/very often: not coordinating team activities, n = 11 (65%); not maintaining the big picture, n = 9 (53%); and incomplete physical examination, n = 9 (53%). Additionally, responses indicated that omission bias, delayed recognition of abnormal vital signs, tunnel vision, lack of prioritizing tasks and missing sensory cues could occur occasionally. External experts suggested additional potential errors: lack of identified team leader, poor crowd control, failure to revise, failure to summarize, failure to perform serial examinations with emphasis on assessment from the initial encounter, over-ordering/relying on tests, over-testing due to liability concerns, poor consultant to consultant interactions, delays in definitive care and lack of ownership. By exploring the cognitive activity of pediatric trauma experts across the United States, we were able to condense their knowledge and experience into a tool for learners (see Table 15 for common pitfalls and Tables 16-19 for comprehensive tools), which we will further explore in the discussion.

**Table 15:** Potential Errors during Management of a Patient with Pediatric Blunt Abdominal Trauma. EMS: Emergency medical services

Management Step	Potential Errors	To Avoid Errors
Pre-arrival	Missing sensory cues	Pay attention to the tone of voice or urgency of EMS (e.g., sirens in the background)
	Lack of prioritizing tasks	Determine if the child is sick or not sick based on the vital signs, the mechanism and obvious injuries
	Not coordinating team activities	Know the activation criteria – post them near the ambulance communication phone
Primary Survey	Not coordinating team activities	Identify team leader and assign roles and equipment tasks
	Lack of prioritizing tasks	Use an organizational approach/bedside aid to assist with equipment sizes and medication dosing for children
	Tunnel vision/fixation/anchoring	Airway/breathing – Recognize the unique anatomy while managing a child’s airway (e.g., larger tongue)
	Missing sensory cues	Circulation – Determine differential diagnoses for tachycardia in a child (e.g., fever, fear, pain, anxiety). Recognize that tachycardia is the earliest sign of shock
	Delayed recognition of abnormal vital signs	Disability – Understand that a child may not be able to cooperate due to age, time of day/poor perfusion, hypoxemia, post-head injury or post-ictal
	Omission bias	Exposure – Lack of full exposure/log rolling a patient may result in missed findings (e.g., bruising)
	Incomplete physical exam	Environment – Children are more susceptible to ambient cooling. Consider warming measures in young children who have higher body surface area to mass ratios
	Not maintaining big picture	Recognize abnormal vital signs and consider potential causes
	Not coordinating team activities	Re-assess the child after every intervention and make clear summary statements, e.g., “this patient is in shock”
	Failure to revise	
Secondary Survey	Incomplete physical exam	Remain systematic in a head-to-toe physical exam, and try to avoid being distracted by obvious injuries (e.g., open wounds)
	Not coordinating team activities	Communicate next steps in management with the team
	Delayed recognition of abnormal vital signs	Use clear summary statements
	Tunnel vision	
	Failure to revise	
Labs, imaging, pain management, disposition	Ordering unnecessary tests	Review the indications and risks (e.g., radiation) vs. benefits before ordering tests
	Not maintaining the big picture	Recognize the unanticipated effects (e.g., change in mental status) from pain management
	Not coordinating team activities	Re-assess the patient after every intervention (e.g., re-assess vital signs and mental status after giving pain medications)
	Lack of reassessment	Use simple clear terms “this patient needs x, y, z” to coordinate patient care
	Failure to revise	Use closed-loop communication with all team members

**Table 16:** Comprehensive Decision-making Tool for Evaluation of Pediatric Blunt Abdominal Trauma: From EMS to Hospital Arrival. ED: Emergency department; EMS: Emergency medical services

From EMS to Arrival at the Hospital				
Management Step	Assessment	Decide	Avoid	Improve
Field triage: Upon EMS call, determine if child is sick based on vital signs, mechanism and obvious injuries	Identify and interpret vital signs/Identify or question circumstances to decide if mechanism raises concern for possible blunt trauma/Identify obvious sources of bleeding	Is there airway obstruction? Is shock present? Is this child sick or not sick?	Missing sensory cues/Minimizing or not obtaining adequate information	Pay attention to the tone of voice or urgency of EMS (e.g., sirens in the background)/Take an organized approach: upon EMS call, determine if the child is sick or not sick based on vital signs, mechanism and obvious injuries
Activate trauma team/process if appropriate	Know the indications for trauma team activation according to the local protocol	How does this patient meet the activation criteria? What level of activation or what staff members are needed to care for this patient?	Failure to prioritize tasks/Undertriage/Asking for too little information	Know the activation criteria/have them posted near the ambulance communication phone
Communicate requirements for staff, equipment and resuscitation	Outline the patient’s care needs/consider available space (e.g., do room assignments need to change?)/Consider any unique requirements	Are there needs for this individual patient such as massive transfusion protocol, extensive warming measures, hemorrhage control? Is there space available? Is room rearrangement required? Are there unique requirements?	Not coordinating team activities/Poor crowd control	
Obtain information from transport about mechanism				
Estimate weight of patient				
Assess if patient is not breathing or pulseless				
Obtain most recent set of vitals from EMS	Identify and interpret vital signs		Failure to prioritize tasks/Tunnel vision	
Transfer patient from EMS gurney to ED bed				
Obtain EMS report				
Go to Primary Survey				

**Table 17:** Comprehensive Decision-making Tool for Evaluation of Pediatric Blunt Abdominal Trauma: First 5 Minutes of Care. BVM: Bag-valve mask; BP: Blood pressure; CBC: Complete blood count; GCS: Glasgow Coma Scale; HR: Heart rate; IO: Intraosseous catheter; LR: Lactated ringers; LFTs: Liver function tests; NS: Normal saline

First 5 Minutes of Care				
Management Step	Assessment	Decide	Avoid	Improve
Determine whether patient meets trauma team activation criteria	Identify life-threatening conditions such as poor perfusion or abnormal mental status (doorway assessment)/If team leader not present, determine leader for any needed resuscitation (until team leader arrives)	How sick is this patient? Who will perform the initial assessment? Who will begin leading the team until this formal role has been filled? What trauma team activation criteria does this patient meet? Doorway assessment upon arrival: Is this patient not breathing or pulseless? (If no information available, begin primary survey)	Not coordinating team activities/No forward steps in care because designated team leader not yet present/Delay in recognizing the need for activation/Delay in starting a resuscitation	Recognize that the child may not clearly meet criteria prior to arrival but that changes in status or physician discretion for need indicate the need to activate/Identify team leader/Make sure handoff to team leader is short and contains salient information
Estimate patient weight	Determine patient’s weight as soon as possible/Weigh the child if stable; if not, use weight-based tape or age-based method to estimate (weight will determine equipment sizes and medication dosing for resuscitation)	What is the patient’s weight? If patient is unstable, how will weight be estimated? How does this method work? How is equipment organized? Is weight-based equipment and dosing available (e.g., in color-coded bag or cart)?	Failure to prioritize tasks/Not determining accurate weight	Recognize that equipment and medication adjustments are unique to pediatric practice/Rather than trying to commit these to memory, implement an organizational approach or bedside aid such as an app or chart/Make sure resuscitation equipment tasks are clearly assigned
Airway assessment	Maintain C-spine precautions/If awake, observe phonation/If unconscious, look-listen-feel/If not breathing, ventilate with bag-valve mask (BVM)/If BVM, evaluate patency/If intubated prior to arrival, note route (oral or nasal) and adjust airway if needed	Does this patient show evidence of life-threatening airway injury? Is there upper airway obstruction? Is the airway midline? Does facial injury make the airway not maintainable? Is neck crepitus present? Is there possible neck injury? Does the head/neck need to be stabilized? Is child responsive enough to maintain airway? Is child breathing primarily through nose (under 12 months old)?	Failure to prioritize tasks/Lack of focus and organization, moving forward from one step to the next/Non-systematic physical examination/Tunnel vision, fixation or anchoring (e.g., distraction of attention due to obvious injuries or bleeding)	Children may have partial or complete airway obstruction due to head injury and/or poor perfusion, with decreased mental status/Children have relatively larger tongues and their airway is more likely to be obstructed by secretions, vomit, candy or food/Frequently practice steps in airway assessment/After each step, decide management/Assign roles to additional staff if available
Breathing assessment	Auscultate two lung fields bilaterally/Inspect equal chest rise/Recognize immediate life-threatening chest trauma	Respiratory arrest: Is the patient breathing? Inadequate ventilation: Is the patient breathing well enough? If not breathing, can patient be bagged with an assist device? If BVM is airway patent? If intubated prior to arrival, is tube size adequate? Is tube in correct location? Is chest crepitus present? Unequal breath sounds? Pneumothorax? Tension? Tamponade?	Failure to prioritize tasks/Failure to recognize poor ventilation	Hypoventilation and hypoxemia can worsen the outcome of any injury, so they must be recognized and managed ASAP/If patient not breathing, death immediate within 5 minutes
Central circulation assessment	Palpate central pulses (femoral or brachial)/Evaluate capillary refill and skin temperature/Evaluate for obvious bleeding/Determine heart rate and blood pressure and apply monitors	Is the patient perfusing? Could patient have life-threatening hemorrhage? Is there evidence of shock? If so, what could be the cause (e.g., hemorrhagic shock, tension pneumothorax, cardiac tamponade)? Does patient need transfusion of blood products? Does patient need massive transfusion protocol activated? Does patient need to be moved to OR for operative stabilization?	Delayed recognition of abnormal vital signs/Failure to understand the meaning of tachycardia in an injured child	Tachycardia is the earliest sign of shock/Children can have significant blood loss (up to 40%) and be capable of compensation, so their blood pressure may be in the normal range/There are many causes of tachycardia other than shock, but it must be explained in the trauma setting (potential causes include acute blood loss, hypoxemia, pain, fear, anxiety or a combination)
Disability assessment	Determine level of consciousness (alert, verbal, painful, unresponsive)/Check size and responsiveness of bilateral pupils/Perform quick four-extremity strength and movement test/Perform Glasgow Coma Score (GCS) assessment	Does child appear awake? Alert? Is patient cooperative? If not cooperative is it because child is shy or afraid? Is child moving arms and legs? What is the GCS? What is child’s normal baseline and/or developmental stage? Is there evidence of severe head injury with possible herniation syndrome?	Omission bias/Failure to recognize that head injuries are very common in childhood	The ability to cooperate changes with age/Children may be sleepy due to time of day, poor perfusion, hypoxemia, post-head injury or post-ictal/Shock is usually not caused by head injury, but shock may present with altered mental status
Exposure environment	Make sure child is fully exposed/Identify any potential sources of bleeding that are visually apparent (thigh deformity, pelvic instability, open wounds)/Log roll patient off any spine board/Begin warming measures	Are there any obvious sources of bleeding? If so, can direct pressure be applied now? Is there evidence of bruising/contusions? If contusions present, are they consistent with history? Is patient at risk due to environmental temperature? What warming measures are available?	Incomplete physical examination/Not maintaining big picture	Lack of full exposure and log rolling patient may result in an incomplete examination and missing important clues (e.g., bruising)/Room temperature in most hospitals will compromise perfusion and children are more susceptible to ambient cooling/Consider immediately beginning warming measures in young children (they have greater surface-area-to-mass ratios than older children and adults)
Family/Facility	Determine actions to address parent or caregiver needs/Determine whether child needs to be transferred according to local capabilities	Should parents be present during resuscitation? What statements will be made? Does your facility have the capability to care for critically injured children? Does child need to be transferred to another facility/department? If so, how will they be transported and by whom?	Not coordinating team activities/Not maintaining big picture/Failure to communicate big picture	
Apply monitoring if not already done so (can be done simultaneously with above assessments)	Perform vital signs as fast as possible, including heart rate, blood pressure, temperature, oxygen saturation	Are vital signs normal or not? Is blood pressure cuff the appropriate size based on age/weight of child?	Failure to prioritize tasks/Missing sensory cues/Failure to revise	Any abnormal vital sign must be explained and potential causes sought/Watch for trends in vital signs, especially heart rate
If a trauma team process is in place and not already activated, does child meet activation criteria?	Match child’s level of acuity to patient care capability at the bedside	How sick is this child? Does patient meet criteria for trauma activation? If there is more than one patient simultaneously, is this patient the sickest?	Failure to revise/Not coordinating team activities	Determine differential diagnoses for tachycardia in a child (such as fever, fear, pain, anxiety)
Make a declarative statement of assessment	Categorize the preliminary assessment and next steps	What is patient’s status up to this point? What has been communicated among team members? What are the priorities?	Not coordinating team activities/Failure to communicate	Team leaders should practice this step/Recorders or scribes should provide prompting questions about patient’s status
Obtain intravenous access	Determine the need (and priority) for vascular access/Clarify route, number of attempts for each route, and number of lines needed	Does patient require vascular access? How soon? How many lines? How many attempts? What routes (IV/IO/central access)?	Not maintaining big picture/Prolonged attempts at IV access	Avoid IO placement in an awake patient without local anesthesia
Obtain laboratory testing	Consider CBC, LFTs, lipase, basic metabolic profile (BMP), type and screen or type and cross, urine dip or urinalysis	What is the rationale for labs? What are the normal values for children of different ages?	Ordering unnecessary tests (e.g., consider whether amylase is indicated)	
Identify need for fluid resuscitation with crystalloid, blood products or massive transfusion protocol	Consider vital signs and perfusion (HR, BP, capillary refill, pulses)	Does patient require volume resuscitation? What rate of infusion? What type of fluid (NS or LR 20 cc/kg)? What type of blood products?	Failure to revise/Delayed recognition of abnormal vital signs and shock	Determine differential diagnoses for tachycardia in a child (such as fever, fear, pain, anxiety)

**Table 18:** Comprehensive Decision-making Tool for Evaluation of Pediatric Blunt Abdominal Trauma: Within First 15 Minutes. BVM: Bag-valve mask; BP: Blood pressure; CT: Computed tomography; FAST: Focused assessment with sonography for trauma

Within First 15 Minutes				
Management Step	Assessment	Decide	Avoid	Improve
Roll patient and assess	Consider that there may be injuries present that were not recognized prior	Are there findings not visible?	Incomplete physical examination	Remove clothing/Remove board from under patient
Consider chest X-ray	Note changes in physical examination that may evolve over time	Is there air outside lung (such as crepitation in the neck or over the chest wall)? Are there rib fractures?	Not coordinating team activities	Note that multiple rib fractures are correlated with increased rate of death
Assign team member to update family and obtain further history	Consider family need for briefing and staff support	Who will be assigned to family? Are family members cooperative?	Not maintaining big picture	
Reassess primary survey abnormalities for improvement	Determine if primary survey derangements have begun to improve	What reassessment parameters are needed for patient’s volume status? Does initial crystalloid volume infused improve the perfusion and vital signs? Does patient show evidence of fluid overload? Is more crystalloid volume needed? Should patient receive blood products?	Not coordinating team activities/Failure to communicate/Missing sensory cues/Failure to revise/Delayed recognition of abnormal vital signs/Failure to reassess and follow up on interventions for improvement or deterioration	
Team leader: Provide repeated assessment stated out loud	Communicate reassessments clearly to keep team moving forward	What is the current status of this patient? Is this status evaluation shared by all team members? Are all team members on the same page in the resuscitation?	Omission bias/Failure to revise/Anchoring on initial assessment/Failure to summarize	Simple, clear terms help: “I am worried about severe head injury” or “This is shock”
Consider pain management	Consider hemodynamic status, weight, past medical history, allergies	Does the patient need pain control now? What are the benefits (e.g., improved heart rate) versus the risks of pain control?	Not maintaining big picture/Failure to revise	Recognize the possibility of creating unanticipated effects, such as change in mental status, breathing or BP from too much narcotic
Secondary Survey (perform a thorough head-to-toe assessment)	Identify and describe findings (e.g., “only flexing arms to pain”)/Findings associated with injury mechanism suggest accident/Findings not explained by history suggest possible inflicted injury	Does child have examination findings to support blunt abdominal trauma? If poor perfusion, hypotension or tachycardia present, what is underlying cause? What was the mechanism of injury? What findings are present: Bruising? Seatbelt sign on abdomen? Pain? Bilious emesis or hematobilia? Abdominal distention?	Incomplete physical examination/Tunnel vision/Failure to revise/Lack of shared mental model (e.g., a novice may not use same model for altered mental status)/Missing sensory cues	While vomiting is common for injured children, it is not typically bilious/Children may not be able to eat or drink with significant duodenal contusions/Distention should be considered significant/Gastric distention may limit lung volumes
Consider FAST examination	Consider evaluating for cardiac tamponade/Consider evaluating for free fluid (however, CT abdomen/pelvis will reveal more information)	Know the indications for FAST examination at your institution: When should patient simply get a CT scan?	Ordering unnecessary tests and delaying care if patient unstable (provider dependent)	
Consider gastric decompression (nasal/oral gastric tube placement)	Assess for gastric distention or difficulty with ventilation	Has child had BVM ventilation for a prolonged period? Has child vomited? Is there evidence of gastric distention?		Children vomit frequently for many reasons, so vomiting alone does not suggest an increased likelihood of significant injury/Consider that bagging may create sufficient gastric distention to limit lung volume

**Table 19:** Comprehensive Decision-making Tool for Evaluation of Pediatric Blunt Abdominal Trauma: Within First 60 Minutes. CT: Computed tomography; ICU: Intensive care unit; OR: Operation room

Within First 60 Minutes				
Management Step	Assessment	Decide	Avoid	Improve
Consider CT abdomen/pelvis	Determine when it is safe to move patient to CT (e.g., if hemodynamically stable)	What are the indications for CT scanning in abdominal trauma in children?	Ordering unnecessary tests/Not minimizing radiation to the child	Review the indications: pain, tachycardia, abdominal bruising, bilious/bloody emesis
Consider pelvic X-ray	Consider pelvic X-ray in a hemodynamically unstable patient if not getting a CT abdomen/pelvis		Ordering unnecessary tests/Not minimizing radiation to the child	
If patient leaves trauma bay for any reason, determine team needs	Ensure clear team roles and needs as patient moves from one clinical area to another (such as from trauma bay to radiology or OR)	Who is managing the patient during this workup?	Not coordinating team activities	
Determine disposition	Determine whether patient requires hospital admission/Determine whether patient can be admitted to a regular medical-surgical inpatient bed versus ICU or OR	Where does this patient need to be admitted (medicine, surgery, ICU, OR)?	Not coordinating team activities/Poor consultant-consultant interactions/Poor ownership of patient	

## Discussion

Proper pediatric trauma stabilization requires provider knowledge, skills, and behaviors that are child-centered. Providers must be prepared to exercise sound clinical judgment and conduct a well-organized initial assessment of the injured child, recognize the patient’s acuity level, and make decisions in a deliberate stepwise fashion to succeed. Community hospital providers particularly experience difficulty maintaining this level of proficiency as they encounter a limited number of pediatric high-acuity situations [21,22]. Providers need to practice decision-making regularly to keep their skills fresh and to maintain confidence.

Currently, many providers at rural or community hospitals are underprepared to deal with pediatric trauma. Even trainees at academic trauma centers may have limited exposure or focused training to become proficient at managing pediatric trauma cases. Emergency care physicians are required to take the ATLS course, but only a small percentage of this course is devoted to pediatrics [13], and consequently, there is a lack of opportunities for providers to practice these skills. Moreover, there is significant evidence that these skills decay over time, and it is expensive and time consuming for providers to travel to these in-person courses [23]. According to recent literature, current teaching methods in PEM are inadequate to properly train providers for infrequent, dangerous events, such as trauma [24]. Other studies have shown that skills related to Advanced Cardiac Life Support (ACLS), a certification for providers, decay within six months, while the course is only taken every two years [25]. Some training facilities attempt to refresh skills with high fidelity simulation, such as mock codes and mannequin training, which require extensive planning and coordination, as well as expensive technology that is generally inflexible to the presence of new training needs and the presence of expert facilitators, thus making it impractical to train providers who have limited time and resources on the latest and most important skills.

As abdominal trauma is the leading cause of unrecognized fatal injury in children, training focused on its management is essential. Most practitioners base their pediatric trauma management on knowledge from prior experiences, medical literature, courses, and advice from experts. Due to limited exposure to pediatric trauma cases, the decision-making skills of a practitioner for a traumatically injured child may not be second nature. Our CTA results may be used to help learners to think like an expert. This CTA provides more explanation for decision-making compared to existing literature on trauma management or courses. While these resources outline the steps in management, they do not explain the associated decision-making with each step. This CTA includes more information, such as sensory cues.

Learners may also turn to experts to ask questions about management of a child with blunt abdominal injury; however, experts may have become unconsciously aware of their automated skills over years of experience [9]. For an experienced practitioner, it may be second nature; however, a novice must synthesize new information to plan next steps and may risk falling into novice traps/potential errors. Automaticity may occur with tasks that are practiced repetitively, such as assigning roles and conducting a systematic primary and secondary survey. Although relying on automaticity to perform skills is efficient, it may interfere with teaching if experts are unable to articulate their thought processes.

To counteract limited education in pediatric trauma, our findings based on national experts in trauma management may be used as a comprehensive supplementary tool to teach decision-making skills (Tables 16-19). It may be used to efficiently teach practitioners about blunt abdominal trauma management by highlighting the key decision-making points and novice traps/potential errors as elicited from experts. The tables outline the steps in the trauma process, associated assessment steps, decisions, things to avoid and suggestions to improve the process for pediatric blunt abdominal trauma management. Many of these decisions are shared by all mechanisms of a traumatic injury until primary and secondary surveys are completed.

Application of our CTA results can effectively decrease training time and increase proficiency for learners by highlighting important decisions outlined by experts. The CTA provides a pathway for practitioners to improve their decision-making skills when managing a patient with pediatric blunt abdominal trauma. These results may also be used as a script for simulation or used to develop an assessment tool or algorithm, for example, creating a decision-making algorithm to teach learners how to effectively make clinical decisions for management of pediatric blunt abdominal trauma. A future study may involve researching the efficacy of such a tool for teaching in a randomized trial and/or test the validity of such an assessment tool. Alternatively, our findings may be utilized as a blueprint for serious video gaming.

Limitations

Using CTA to create such a framework is novel to the field of pediatrics, emergency medicine, and specifically trauma management. Moreover, management of blunt abdominal trauma is a more dynamic process compared to prior studies that applied CTA to procedural and surgical skills. Although we had a small sample size of seven for our CTA, we believe data saturation was ensured through a purposive/theoretical sampling of experts in PEM and pediatric trauma surgery and by gaining a comprehensive understanding of the dimensions of the concepts and themes that emerged from the CTA. Watling and Lingard [26] argue that data saturation is easier to achieve with a theoretical sampling than a convenience sampling. Furthermore, our results were strengthened by external validation of our study through a nationwide survey of experts from PEM and pediatric trauma surgeons. We found a lack of national data on the use of FAST examinations for blunt abdominal trauma in children, highlighting the need for future studies in this area.

## Conclusions

CTA may be applied to the management of pediatric blunt abdominal injury to outline the clinical decision-making process and more specifically to identify critical steps and potential errors to serve as a framework for education in PEM and pediatric trauma surgery. This tool can help train learners to think like an expert and it will also allow experts to share key teaching points with trainees. The findings from this study may be used as a script for simulation or serious video gaming. Future studies may apply CTA to other types of traumatic injuries and/or involve an interdisciplinary approach.
